# Who benefits from a shouldice repair?

**DOI:** 10.1007/s10029-024-03159-w

**Published:** 2024-09-06

**Authors:** Christoph Paasch, Marguerite Mainprize

**Affiliations:** 1 Department of Surgery, Shouldice Hospital, 7750 Bayview Ave, Thornhill, ON, L3T 4A3 Canada; 2 Department of General Surgery, University Hospital Brandenburg an der Havel, Brandenburg Medical School Theodor Fontane, Brandenburg, Germany

Dear Editor,

According to the 2018 HerniaSurge guidelines, the Shouldice Repair is considered the best non-mesh suture repair for inguinal hernias [1, 2] and has been the *strongly* recommended non-mesh repair [1]. The 2018 guidelines also stated that “*the use of open non-mesh repair in specific patients or types (e.g. young males with lateral hernia L1 and L2) of inguinal hernia as an acceptable alternative to a mesh technique has not been adequately investigated so far.”* [1]. However, at the end of the guidelines there were recommendations for future research in this area due to limited available data. This research included randomized controlled trials or propensity score matching.

In 2018 Köckerling et al. used the German Herniamed registry to perform a propensity score matching study, comparing the Shouldice Repair (*n* = 2608) to the Lichtenstein (*n* = 2115), TEP (*n* = 2225), and TAPP (*n* = 2400) procedures [3]. Patients who received the Shouldice Repair were mostly young (mean age: 40 years) males with a mean Body Mass Index of 24 kg/m^2^, and 85% of the defect sizes were not larger than 3 cm. This analysis revealed comparable one-year results between the Shouldice Repair and the Lichtenstein, TEP and TAPP procedures [3]. Based on the Kockerling et al. findings the HerniaSurge group stated in the 2023 updated guidelines that *“In selected groups of patients with primary unilateral inguinal hernia repair*,* the Shouldice technique achieves one-year outcomes comparable to that of Lichtenstein*,* TEP and TAPP operations providing expertise and competence are available”* [2].

However, four of the Shouldice Hospital’s recently published high-volume studies [4–7] provides data that appears to be comparable to published high-volume registry data on mesh repair [3, 8]. From these four studies there were 27,751 patients who underwent surgery for groin (> 95% inguinal) hernias between 2013 and 2024 [4–7]. The mean age of these patients ranged from 56.2 years (61–85; *n* = 25785 [4]) to 62 years (51–70; *n* = 1120 [6]) and the average Body Mass Index was 25.0 kg/m^2^. Two of the publications provided ASA information, with most patients having an ASA-Score of II and III [6, 7]. Approximately one third of the patients had a large hernia [4, 6]. One publication revealed a recurrence rate of 1.6% after a median follow-up of 16.5 months (*n* = 1120) and these recurrences were assessed by phone calls [6]. The findings are summarized in Table 1. They are also comparable to those of high-volume Herniamed registry data on the Lichtenstein procedure (*n* = 10.555; Body Mass Index: 25.7 kg/m^2^ (± 15.6); Age 63.2 years (± 15.4); one-year recurrence rate 0.83%) [8].

**Table 1:** Publications on groin hernia repair at the Shouldice Hospital in comparison to Herniamed data

Author/Year	Study *N*	Study Type	Study Period	Mean Age (years)	Body Mass Index (Kg/m^2^)	ASA	Primary Objective
Spencer Netto/2023	25,785	Retrospective	2013–2017	56.2 (61–85)	25.0 (14.5–42.9)	NA	Temporal pattern of recurrence
Mainprize/ 2023	414	Prospective	2019–2020	57.5 (18–86)	25.1 (17.7–31.9)	NA	Incidence of opioid use and pain
Paasch/ 2024	1,120	Prospective	2021–2023	62.0 (51–70)	25.2 (23.6–26.6)	I: 25.3% II: 39.7% III: 34.4%	Recurrence rate
Mainprize/ 2024	500*	Prospective	2023–2024	61.1 (25–89)*	24.9 (16–34)*	I: 25.2%* II: 43.0%* III:31.8%*	Patient reported outcomes
Kockerling/2018	2,600	Retrospective	2009–2015	43.7 ± 20.0	24.2 ± 3.5	NA	Patient reported outcomes

*68 cases of ventral hernias included

With this in mind and in contrast to the current guideline recommendations it is possible that even older patients with a higher BMI and more concomitant diseases could benefit from a Shouldice Repair.

## Electronic supplementary material

Below is the link to the electronic supplementary material.

Supplementary Material 1

Supplementary Material 2

## References

[1] HerniaSurge Group (2009) International guidelines for groin hernia management. Hernia 22(1):1–165 10.1007/s10029-017-1668-x PMC5809582 29330835

[2] Stabilini C, van Veenendaal N, Aasvang E, Agresta F, Aufenacker T, Berrevoet F, Burgmans I, Chen D, de Beaux A, East B, Garcia-Alamino J, Henriksen N, Köckerling F, Kukleta J, Loos M, Lopez-Cano M, Lorenz R, Miserez M, Montgomery A, Morales-Conde S, Oppong C, Pawlak M, Podda M, Reinpold W, Sanders D, Sartori A, Tran HM, Verdaguer M, Wiessner R, Yeboah M, Zwaans W, Simons M (2023) Update of the international HerniaSurge guidelines for groin hernia management. BJS Open 5;7(5). 10.1093/bjsopen/zrad080 PMC10588975 37862616

[3] Kockerling F, Koch A, Adolf D, Keller T, Lorenz R, Fortelny RH, Schug-Pass C (2018) Has Shouldice repair in a selected group of patients with inguinal hernia comparable results to Lichtenstein, TEP and TAPP techniques? World J Surg 42:2001–2010 29299648 10.1007/s00268-017-4433-5 PMC5990577

[4] Spencer Netto FA, Paasch C, Yilbas A, Degani C, Svendrovski A, Szasz P, Mainprize M (2024) Temporal patterns for inguinal hernia recurrence operations after Shouldice Repair. Hernia 28(2):607–614 38280050 10.1007/s10029-023-02955-0

[5] Mainprize M, Yilbas A, Spencer Netto FAC, Svendrovski A, Katz J (2023) Incidence of opioid use and early postoperative pain intensity after primary unilateral inguinal hernia repair at a single-center specialty hospital. Langenbecks Arch Surg 408(1):366 37726600 10.1007/s00423-023-03111-z

[6] Paasch C, Mainprize M, Hunger R, Spencer Netto FNS (2024) Polypropylene vs. stainless-steel Wire suture: short-term recurrence rate after Shouldice primary inguinal hernia repair, a non-inferior analysis among 1120 patients a case-control study. Hernia Epub ahead 10.1007/s10029-024-03110-z PMC11530496 39210196

[7] Mainprize M, Paasch C, Svendrovski A, Spencer Netto FAC (2024) Patient reported outcome: pilot analysis of 500 patients. Br J Surg 111:5

[8] Köckerling F, Stechemesser B, Hukauf M, Kuthe A, Schug-Pass C (2015) TEP versus Lichtenstein: which technique is better for the repair of primary unilateral inguinal hernias in men? Surg Endosc 30(8):3304–3313 26490771 10.1007/s00464-015-4603-1 PMC4956717

